# A Novel Practical Session to Teach Concepts of Allometric Scaling of Brain Structures to Undergraduate Students Using Vertebrate Brains

**DOI:** 10.59390/001c.154559

**Published:** 2025-12-31

**Authors:** Christopher Cammies, Kelly Cavaciuti, Stephen H. Montgomery

**Affiliations:** 1 School of Biological Sciences University of Bristol

**Keywords:** retina, critical thinking, anatomy, brains, allometric scaling, lab activity

## Abstract

Scaling relationships are central to interpreting patterns of morphological variation in brain composition. However, allometric scaling can be a difficult concept for students to understand, requiring the integration of evolutionary biology with mathematical relationships. The differential scaling of brain components over evolutionary time is particularly complex. The challenges associated with these concepts are further compounded by the lack of practical activities to allow students to explore these concepts in a neuroscience context. In this study, we present a novel practical session to teach these ideas to second year biology and zoology undergraduate students by combining traditional sheep and pig brain dissections with accessible staining techniques, and imaging using freely available software, that together enable allometric scaling relationships among brain components to be visualised and analysed in both an intraspecific and interspecific manner. Objectively, our data shows a statistically significant improvement (p=<0.0001) in performance on questions related to the scaling concepts following the practical session. Subjectively, 93% of students wanted the lecturer to continue teaching this practical (with 0% of students against it being reused in future), with 89% believing the practical had increased their interest in studying neuroscience. Most students believed the practical had improved their understanding of the concepts and enhanced their ability to critically analyse literature on the topic of allometric scaling and brain anatomy. Students’ perceptions of the practical were positive with the average rating of perceived learning 8.11 out of 10 (where 10 is an excellent learning experience and 1 is a terrible learning experience). Aside from minor technical suggestions, the main improvements suggested by students were that they wished they had more time for the practical.

Neuroscience education should ensure students have suitable subject knowledge and understand key concepts but also provide students with ample opportunities to develop critical thinking skills. Critical thinking encompasses the application of knowledge, the ability to analyze data, evaluate the rigor and quality of data and methodologies and the ability to create new ideas and concepts. These critical thinking skills are known as higher order cognitive skills (HOCS) (Zoller, 1993). Existing literature suggests that active learning is better for the development of HOCS compared to more passive learning modalities such as lectures (Harris & Bacon, 2019; Kusumoto, 2018; Styers et al., 2018). Unfortunately, due to the complicated equipment and methodologies involved in neuroscience, many concepts can be challenging to teach in an active, practical and engaging manner. However, innovative and relatively low-cost practical sessions have been made to actively teach complicated topics, such as neuroscience research methodologies that involve the development of critical thinking skills (Segawa, 2019).

Scaling relationships between brain components can be a particularly challenging neuroscience topic to teach due to the integration of evolutionary biology with statistics and mathematics. Many aspects of brain architecture are the result of allometric scaling, whereby the size of each brain region is predicted by variation in overall brain size, through a classic log-log relationship, *log(y) = βlog(x)+ α* (where y is the size of a region of interest, x is a measure of overall brain size, β is the scaling coefficient (slope), and α is the intercept). However, allometric scaling coefficients can vary between structures, with some showing negative allometry (hypo-allometry, scaling coefficient <1) and others showing positive allometry (hyper-allometry, scaling coefficient >1). This means that as a proportion of brain size (i.e. size expressed as a percentage of overall brain size) some structures appear disproportionately reduced or expanded when comparing large brains to smaller brains. For example, differences in the proportions of myelinated axons, and therefore white matter, lead to variation in scaling coefficients between the cerebellum and neocortex, with the neocortex increasing in size more rapidly with increasing brain size (Bush & Allman, 2003). This is important, as discounting variation in allometric scaling can conflate interpretations of how groups deviate from expected patterns, for example when trying to understand human-specific neural traits (Montgomery, 2013). Similarly, variation in the scaling intercept, α, which reflects non-allometric scaling (also referred to as ‘grade-shifts’), is commonly used to understand variation in brain composition across species that may be caused by adaptive evolutionary processes, so is critical to evolutionary neurobiology (Montgomery et al., 2016). Understanding and critiquing analysis of brain scaling is therefore of primary importance for comparative neuroscience.

While data interpretation questions are freely available in the general area of allometry (which could be easily modified to a neuroscientific context) and there is an excellent example of active learning with students generating and analyzing data using online images of different brains (Grisham et al., 2018) other examples where students could do the physical laboratory work are lacking. We therefore set out to create a practical session using readily accessible materials and simple methodologies whereby undergraduate students could generate their own data to explore ideas relating to scaling of brain structures and develop both knowledge and critical thinking skills.

We developed a practical based on exploring brain scaling in vertebrates. Sheep and pig brains are common waste products from the meat production industry and are rarely sold as food. These brains are, however, commercially available from science education companies, butchers and local abattoirs at relatively low prices. Using dissections, a modified version of the Mulligan’s technique (Mulligan, 1931) and freely available imaging software, FIJI (Schindelin et al., 2012), in our practical students can explore the masses of different brain structures and stain and measure the volume of grey and white matter present within some of these brain structures. By pooling class data, they can explore how brain scaling relationships vary with increasing brain size and compare inter-specific and intra-specific variation. In particular, the divergent sensory ecologies of sheep and pigs lead to the prediction that they may invest differently in the olfactory lobe, providing a context to explore group differences in the intercept (α) using structure masses, while expected differences in the proportions of white and grey matter in the cerebellum and neocortex provide a context to explore group differences in scaling coefficients (β). By basing the practical around these two hypotheses, we can discuss i) general brain structure, ii) the structure of the olfactory system, iii) the impact of brain connectivity, and long-range myelinated axons, on white and grey matter, and iv) core principles of scaling analysis.

To evaluate the effectiveness of this practical we recruited 2^nd^ year Biology and Zoology students on the optional “Neuroethology” unit to do pre and post session surveys including an assessment of their understanding of neuroscience concepts before and after the session, and some survey questions exploring their perceptions of the session as a learning experience. The practical session was preceded by two introductory lectures on brain evolution and included introductory slides on scaling relationships.

## MATERIALS AND METHODS

### Participants, Recruitment and Data Selection

The participants were made up of two cohorts of 2^nd^ year biology and zoology students (74 students in spring 2024 and 125 students in spring 2025), who had self-selected the Neuroethology optional unit (students must select 3 units from a choice of 6). There are no prerequisites for selecting the unit, as all year 1 students cover some basic neurobiology (lectures covering neurons, synapses etc., and a practical involving extracellular recordings of action potentials). No form of gender information was recorded. Students were incentivised to participate in the study with entry into a prize draw for 4 x £25 love2shop vouchers if they filled out two optional surveys (Appendix 1). One survey was completed before the practical, assessing their understanding of a series of neuroscience concepts, and the second survey was completed after the practical session re-assessing their understanding of the same neuroscience concepts, followed by questions capturing their subjective opinions on the learning experience. Ethical approval for the project was granted on 10.1.2023 by the Faculties of Life Sciences and Science Research Ethics Committee at the University of Bristol. A total of 131 of the 199 students filled out survey one (response rate of 65.8%) and 73 students completed the second survey (overall response rate of 36.7%).

### Investigating Scaling Relationships in Vertebrate Brains: Session Details

Students enrolled on the Neuroethology unit were encouraged to watch a pre-recorded video covering content related to anatomical terms (such as rostral, caudal, medial, lateral etc.,) the anatomy of different brain structures (hippocampus, thalamus, etc.,) and some of their reported functions. When they arrived at the session, they had a handout (Appendix 2) and a hemisphere of either a sheep brain or pig brain (between a pair of students). They were encouraged to locate various structures on the brain and label relevant diagrams on their handout.

Students were then didactically introduced to the learning objectives and structure of the session; in essence they would compare the masses of different brain structures relative to total brain mass within a species (sheep or pig), and then again between species, and test hypotheses about how brain structure varies within and between the species. In particular, they test the hypothesis that the olfactory bulb is non-allometrically expanded in pigs, reflecting an increased olfactory sensitivity, and resulting in a ‘grade-shift’ between the species (H1) (Schild & Rørvang, 2023). The students would then slice and stain the neocortex and cerebellum of their hemisphere and compare the scaling relationship between grey and white matter in the different brain structures, both within and between species. Here, they are predominantly testing the hypothesis that white matter scales more steeply with grey matter (has a higher scaling co-efficient) in the neocortex, compared to the cerebellum, due to a greater proportion of myelinated, long-range axons (H2) (Barton, 2012; Bush & Allman, 2003).

Students were shown how to dissociate the neocortex, olfactory bulb, and cerebellum, leaving the remaining brain (coded “rest of the brain”) and how to record the mass of each. All students did this and entered their data (from one hemisphere only) into a shared spreadsheet (a code unique to each animal was provided so they could link their hemisphere with the group who measured the corresponding hemisphere for that brain). The class data was pooled using this spreadsheet, averaging values of paired hemispheres where available. A comment text box was included in the form for students to record observations of structure damage or method error, and this assisted the removal of obvious outliers due to student error. Using log-transformed data students explored variations in total brain mass, brain structure masses, and differences between the species. They then compared the mass of each brain structure to that of the “rest of the brain” mass, which was used as the allometric control (independent variable) as total brain mass would have statistical issues with auto-correlation using linear regressions. This allowed the students to explore scaling relationships (e.g. how does olfactory bulb mass scale with increasing brain mass within sheep) in real time and get timely feedback from academics running the session on the concepts.

After the exploration of scaling relationships in the masses of different brain structures, students were shown how to manually section and stain the neocortex and cerebellum such that the grey and white matter could be visualized. Student instructions can be found in Appendix 2 with a technical prep sheet found in Appendix 3. Once they had stained their sections, they took high resolution photographs and used FIJI (Schindelin et al., 2012) to calculate the surface area of grey and white matter for each section and multiplied this by the depth of their section(s) to get an approximation for the volume of grey and white matter. This was then entered into a class dataset such that students could explore the scaling relationship between white and grey matter both within and between species with an expanded dataset. This provided another real-time opportunity to analyze data (with academic support on hand) and give students the opportunity to think critically about scaling relationships.

The session finished with students being encouraged to evaluate the limitations of the methodology and come to conclusions about the scaling relationships explored in the session. The academic leads brought together some of these ideas, dealt with some misconceptions and summarized the key ideas covered in the session. At this point students were encouraged to consider completing the optional post-session survey. In the interest of time, students initially explored the data using Microsoft Excel in the practical session itself but were provided with resources to use formal regression models using the SMATR package in R (Warton et al., 2012), for their formal practical report which followed the practical sessions. This formal report included writing 500-word sections on two focused tasks relating to the statistical analysis of scaling relationships, and the creation of relevant figures. The report was summative, with a deadline two weeks after the practical, and provided practical leads with an insight into the ability of the students to collect and analyze data.

### Quantitative Analysis

To test if the session enhanced student understanding of the underlying neuroscientific concepts involved, participants completed a survey at the start of the session that included 5 multiple-choice questions (survey questions 4, 6, 8, 9, 11) that were only covered in lectures delivered before the practical (if they attended them) and 5 multiple choice questions (survey question 3, 5, 7, 10, 12) that had been covered in lectures but would also be covered during the laboratory session (Appendix 1). They would encounter the exact same questions in the survey at the end of the session. This mixture of lecture only and laboratory +lecture content was designed to gauge improvement from the session itself, rather than from discussion of the questions, practice or other factors unrelated to the session. Student data was anonymous and as such they had to use a self-created code to link the pre and post responses together. A total of 131 students filled out survey one (response rate of 65.8%) and 73 students completed the second survey (overall response rate of 36.7%). Only 69 of the 73 responses could be used for the quantitative analysis as four students post-survey data could not be paired with the pre-survey due to a failure of the participants to record the code linking the two surveys together. The differences between pre and post session performance were analyzed using a Wilcoxon-pair-signed-rank test using RStudio (Posit Team, 2025).

### Qualitative Analysis

Multiple-choice questions while effective at assessing knowledge are typically poor at assessing HOCS (Crowe et al., 2008; Gormally et al., 2012; Masters et al., 2001). Multiple-choice questions also provide no information about students’ perceptions of the learning experience. To capture students’ perceptions of the laboratory session with regards to interest, development of HOCS, and as an overall learning experience, the post-session survey included 6 Likert-scale questions, an opportunity to rate learning out of 10, and 3 open-ended questions (see Appendix 1). The questions related to the building of HOCS also had questions which were essentially the inverse of a previous question (e.g. Questions 13.1 and 13.2 which explore whether the practical helped them critique and evaluate literature) to minimize issues with the framing of the questions impacting student perceptions of their progress. Across the two cohorts surveyed in this study we had 73 responses to the post-session survey, and these were all used for qualitative analysis.

### Technical Set Up and Equipment

A full list of equipment and how to set up the session can be found in Appendix 3. This makes the most sense in conjunction with the student instruction in Appendix 2. To extract brains from animal skulls please see full details in Appendix 4.

## RESULTS

### Student Success in Data collection

**Figure 1.**
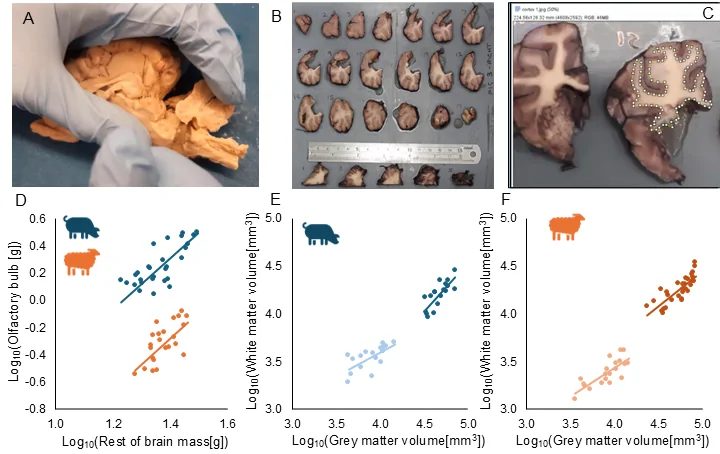
Student led data: A) Photograph of a student dissection of the cerebellum as part of the mass-focused exercise; B) Photograph of sectioned and stained brain (hemispheres) including a ruler for scale in FIJI ; C) Screenshot of segmenting the white matter volume in a stained section using FIJI - ImageJ; D) Example of class dataset of pig (blue) and sheep (orange) olfactory bulb mass, scaled against the rest-of-the-brain, illustrating non-allometric differences in the size of the olfactory bulb; E) and F) Examples of class datasets on white and grey matter volumes in pigs (blue) and sheep (orange) for the neocortex (darker shade) and cerebellum (lighter shade).

Students in the practical successfully collected two pooled datasets, one on brain structure masses, and one on white and grey matter volumes. Students were generally able to analyze these data as instructed, using linear regressions to test for group differences in scaling coefficients and intercepts. Examples of the students conducting the practical work and the pooled data they generated (with obvious outliers removed) are provided in Figure 1.

### Underlying Neuroscience Concepts

**Figure 2.**
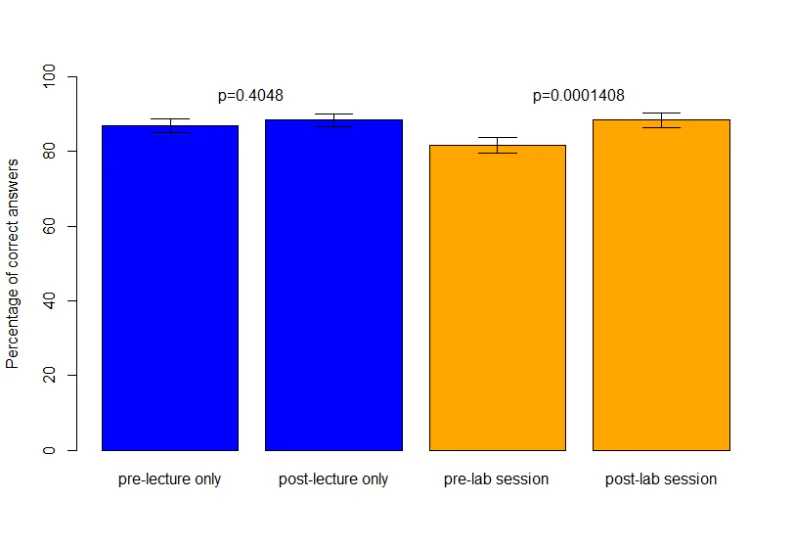
Students answered significantly (determined by Wilcoxon-pair-signed rank test) more questions based on content featuring in both lectures and the laboratory session correctly after the lab session (v=67.5, p<0.001), whereas questions related to content only featured in lectures did not significantly differ after the laboratory session (v=145,p=0.4048). Error bars indicate standard error.

To determine whether the session had led to a significant improvement in the desired neurobiology content taught, a Wilcoxon-pair-signed-rank test was used to compare between answers to questions relating only to lecture content and between questions relating to lecture and the laboratory session content (Figure 2). There was no significant improvement in lecture only content (v= 45, p=0.405) with a slight increase in correct responses from 87% (2 dp) to 88.41% (2 dp). Whereas there was a significant improvement in content covered in the laboratory session (v=67.5, p<0.001) with mean correct responses rising from 81.74% (2dp) to 89.28%.

### Student Perceptions of the Practical Session

**Figure 3.**
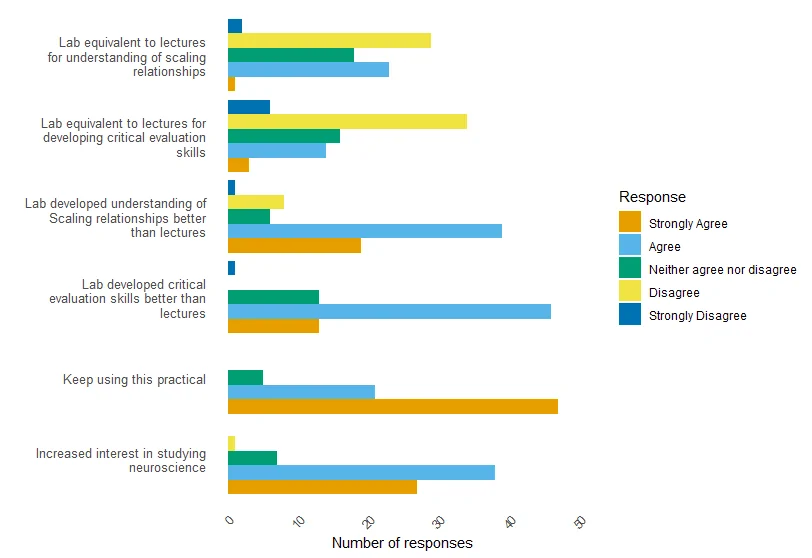
Student perceptions of the practical session in response to abridged summaries of the questions listed on the left, with 5 possible responses ranging from strongly disagree to strongly agree.

Student perceptions of the practical were overwhelmingly positive. Given the opportunity to rate the practical as a learning experience (where 10 is “excellent” and 1 is “terrible”) the mean score was 8.11 out of 10, suggesting that students perceived this to be an effective learning experience. Student responses indicate that the majority of students felt the session enhanced their interest in neuroscience, strengthened their understanding of the concepts involved, and helped them develop HOCS in that topic area (Figure 3).

A clear majority of students (80.8%) agreed that “collecting and analyzing data on vertebrate brains during the practical has helped me to critique and evaluate the findings of other researchers investigating scaling relationships in brains (for example identifying limitations in their methodology) better than if I had only learned about this in lectures?” with only 23.3% of students agreeing with the inverse statement that “I would have been able to critique and evaluate the underlying weaknesses in published studies exploring scaling relationships in brains just as well if I had only learned about this in lectures”.

“Collecting and analyzing data on vertebrate brains during the practical has helped me to understand how different scaling relationships (for brain structures versus total brain size) between species might indicate adaptive shifts in brain structure better than if I had only learned about this in lectures?” was the view point of 79.4% of students with 32.9% of students agreeing with the inverse statement “I would have understood how different scaling relationships (for brain structures versus total brain size) between species might indicate adaptive shifts in brain structure just as well if I had only learned about it in lectures.”

When asked whether covering this topic as part of a hands-on practical had increased their interest in studying neuroscience 89.1% of students agreed, with 93.2% of students agreeing that the lecturers should use this practical for future students.

Further to the Likert-scale questions, students were given the chance to justify their rating of the practical session as a learning experience. While we did not perform a full thematic analysis (Braun & Clarke, 2012) the responses were very positive, and we have chosen a few statements that we feel reflect the broader responses of the students (we have merged some of the constructive feedback in these justifications with responses to the other questions below):

*“Really interesting and the first time I have ever been able to look at a real brain. All the techniques were brand new, and the work felt meaningful.” –* 36 out of 73 student mentioned that the session was fun or interesting. With 3 commenting it was the best practical session of their degree.

*“It helped a lot to visually see the brain to understand the regions and grey and white matter more clearly. It is often difficult to grasp the scale of the brain when just looking at images in lectures.”* – 8 students mentioned this helped them visualize the concepts taught in lectures

*“The instructions were clear, and it was very engaging. There were many aspects involved with the practical, such as dissecting, staining, and digital work including making graphs and working out area of the brain slices. There was a good balance of working on computers and hands-on work. Many new skills were explored too, such as using new software (Fiji J), staining and rinsing using the Mulligan’s technique etc. And most importantly of all, it was enjoyable because it was brains!” - 9* commented positively on the range of skills and techniques covered in the session.

*“It cemented what I learnt about scaling in brains, I hadn’t really understood it before”* -9 students explicitly described an improvement in their understanding in their feedback.

Students were asked to suggest improvements, and give any other thoughts or comments, we have merged these suggestions with constructive feedback from the justification of the learning experience rating and grouped them into 3 main suggestions:

1. Students wanted more time, they enjoyed the session but wanted more time with the brains and exploring the data, some students felt the session was a bit rushed. (n=14 out of 73)
2. A small proportion of students found the imaging of brain sections using FIJI was a bit tedious as they had to repeat it for multiple sections. (n = 3 out of 73)
3. Positive suggested improvements indicating enthusiasm for the session such as requests for more additions, such as pre-prepared brains in jars, different animal brains, and larger datasets to analyze. (n=6 out of 73)

## DISCUSSION

The session description (see earlier in manuscript), accompanying student handout (Appendix 2), technical prep sheet (Appendix 3), and brain dissection guide (Appendix 4) should provide a structure and template for academics wishing to provide a hands-on practical session that enables students to explore scaling relationships among brain components, in particular in vertebrate brains.

Students worked in pairs to dissect, measure, stain and record a range of information about their sheep or pig brain hemi-sphere. When combined with class data students could analyze and interpret the data to build on their fundamental understanding of the neuroscience concepts involved but also to develop and refine their HOCS. Our pre-session data indicated a sound understanding of the concepts involved based on their previously encountered lecture material, but this was significantly improved following the hands-on laboratory session. The lack of improvement in neuroscience content unrelated to the practical session provides evidence that the improvement was not due to peer-peer conversations during the practical and/or searching for specific answers online during the session. We were not able to actually assess whether they had improved in their critical thinking, so whether this perception translates to an actual improvement in HOCS is unknown. However, student perceptions indicate that they believe the session developed their HOCS which is positive, as students who perceive that they have been provided an opportunity for deep learning are more likely to recognize that a session contributed significantly to their learning (Cammies et al., 2024). Students who shared their perceptions indicated a strong enjoyment of the session and, encouragingly, showed an increased interest in neuroscience.

In general, students implemented the methodology to a sufficient standard that the class data only needed some minor quality control before it was ready to use, and some of the trends which we would expect regarding adaptive evolution of brain structures, and changes in white and grey matter volume with increasing brain volume were evident from simple analyses. The session itself prompted many students to ask questions regarding the reliability of the methodology (e.g. regarding section thickness, number of sections analyzed, differential staining between groups etc.,), highlighting the opportunity they had for critical reflection on scientific practice, and there was a strong culture of curiosity throughout the session.

There are, however, some considerations for future session leads. Previous, less refined attempts at the session have shown us that the data generated can be noisy if care isn’t taken with the dissection, so training is important. Of particular note during practical preparation, is that the olfactory bulbs are relatively easy to damage, particularly in sheep where they are smaller, when removing the meninges. This can impact the data collected on structure masses. In the second part of the practical, the number of sections analyzed can also make a big impact on data quality but analyzing every single section image can be boring for some students, so the implementation is a trade-off between data accuracy and student engagement. We currently opt for analyzing every third section in the neocortex and every section in the cerebellum, but more sections would likely improve data quality and consistency. However, it should also be noted that variation in the accuracy of students’ sectioning and staining will mean some noise is unavoidable. However, noisy data does present some learning opportunities and so for our application we were content to sacrifice some data quality to keep the sessions shorter and more engaging.

The session we ran was 3.5 hours (but did factor in time for surveys to be completed) and we do wish we could have spent more time helping students with the analyses in the session. Some students also suggested they wanted a longer session. We personally would’ve enjoyed a morning session of 2-3 hours focused on anatomy, dissection and brain structure mass, and second (after lunch) session of 2-3 hours focused on staining, imaging, and analysis of grey and white matter volumes. This would have allowed for a more leisurely pace, more time on HOCS and more time with students, but unfortunately our current timetable can’t accommodate this. While in future we won’t be surveying the students (which will free up some time) we will still be looking into ways to streamline and optimize the methodology and the session to allow more time to be spent on data analyses and evaluation.

It is also worth noting that both pigs and sheep belong to the same order, the artiodactyls, and perhaps more prominent differences could be detected using different mammalian orders, such as common laboratory rodents, if the brain material is available. We note, however, that the pig and sheep brains are a comfortable size for the dissection skills of inexperienced undergraduates. Alternatively, the session or subsequent assignments (formative or summative) could also use available online images of other mammalian brains like previous studies (Grisham et al., 2018).

The session lead should also consider religious objections to pig, sheep or animal use, and any possible animal welfare objections. We had a few questions about animal welfare, but students appeared satisfied by the justification that these were waste products from the meat production industry, and we emphasized the goal of using material that would otherwise be wasted for a positive purpose. Conscientious objectors to our session in the past have been provided with images, videos, guided online tutorials to work through and a digital version of the practical, and have then completed the data analysis tasks with the rest of the group.

While the sample size for this study isn’t large, and we didn’t directly measure improvements in HOCS, we believe we present compelling data that teaching scaling relationships in this practical format improves student understanding of the related concepts, provides numerous opportunities to develop HOCS, and was a really enjoyable, interesting and engaging practical for the vast majority of students. We would consider it an adaptive “grade-shift” in our own teaching evolution.

### Address correspondence to:

Mr. Christopher Cammies, School of Biological Sciences, Life Sciences Building, University of Bristol, 24 Tyndall Avenue, Bristol, BS8 1TQ, UK. Email: c.cammies@bristol.ac.uk

AND Dr Stephen Montgomery, *School of Biological Sciences, Life Sciences Building, University of Bristol, 24 Tyndall Avenue, Bristol, BS8 1TQ, UK*

Email: s.montgomery@bristol.ac.uk

## Supplementary Material

Appendix 1

Appendix 2

Appendix 3

Appendix 4

Student Survey (Part I)

Student Survey (Part II)
