# Supplementary material for: A Novel Practical Session to Teach Concepts of Allometric Scaling of Brain Structures to Undergraduate Students Using Vertebrate Brains
Source: J Undergrad Neurosci Educ. 2025 Dec 31;24(1):38–46. doi: 10.59390/001c.154559 (PMC13127676; doi:10.59390/001c.154559)
Supplement: Appendix 2 [file junejournal_2025_24_1_154559_322906.docx]

**Appendix 2: Student Handout**

**Investigating Scaling Relationships in Brains**

**Background**

The aim of this practical is to investigate intraspecific and interspecific scaling relationships within and between sheep and pig brains. You will also learn the main features of a typical mammalian brain and be able to compare these brain structures across species. We will do this by comparing the relative and absolute sizes of different brain structures, and changes in the proportion of white and grey matter in brains of different sizes.

To do this you will be provided with both formalin preserved sheep and pig brains which have been produced by dividing the brain sagittally along the midline. This allows the external features (and some internal features) of the brain to be examined.

To see the remaining internal structures and measure the volumes of white and grey matter, you will make transverse sections of the brain. These sections will then be stained to differentiate between the white matter (myelinated axons) and the grey matter (neuron cell bodies and dendrites). Once you have generated these data you will be able to compare the volumes of the grey and white matter in different brain structures in brains of different sizes and species. By using carefully selected graphs and statistical tests you can seek to elucidate the scaling relationships we are interested in.

These approaches are important in identifying evolutionary changes in brain structure, and variability in brain structure that is not explained by changes in overall brain size.

**Concepts Check: Scaling Relationships**

To examine scaling relationships, it is best to begin by consolidating our understand of the terminology and concepts often used to describe scaling relationships. Please use the introductory talk and lecture materials on blackboard to define the following:

**Isometric Scaling:**

**Allometric Scaling:**

When a scaling relationship departs from common allometric scaling patterns that are observed in other species it may indicate where natural selection has driven an evolutionary change in brain morphology, which likely reflects behavioural or sensory adaptation.

**Brain Morphology**

You will need to understand some simple anatomical terms which are listed below, complete the missing definitions:

**Rostral** _____________ **Caudal** _____________

**Dorsal** back or top side **Ventral** belly or bottom side

**Lateral** _____________ **Medial** _____________

**Transverse** across long axis **Longitudinal** along long axis

**Sagittal** _____________ **Coronal.** _____________

What are the membranes surrounding the brain called, and what do they do?

Name the four lobes of vertebrate brains and give a very brief outline of their role.

1)

2)

3)

4)

Examine the **medial cut surface** in figure 1 and identify the labelled structures.

Label on figure 1: spinal cord, cerebellum, medulla, pons, hypothalamus,

optic chiasm, inferior colliculus, olfactory tract, cerebral cortex.

**Figure 1: Medial view of the left hemisphere of a sheep brain**

**Measuring and Comparing Brain Structure**

Compare the external anatomy of the sheep and the pig brains, note any qualitative differences you observe. *Note some differences between specimens maybe due to damage to the brains during collection, transportation, and/or the removal of meninges.*

Measure the mass of the total brain, cerebellum, neocortex, and olfactory bulb and calculate the “rest of the brain” mass (mostly mid-brain and brain stem) by subtracting these values from the total brain mass. Download the relevant spreadsheet from blackboard and add your data. Then use this expanded dataset to answer the following questions.

Using the raw mass:

1. Quantify the intra-specific variation in brain size for each species; do the brains collected (which were all approximately the same age at slaughter) vary in the average mass and range, and if so by how much?

1. In terms of absolute masses are there any detectable differences between the brains of the pig and sheep? If there are any differences, can you hypothesise a biological reason for these?

Next, examine the scaling relationships using log-transformed masses:

1. How does the intraspecific scaling relationship between each of the cerebellum, cerebral cortex and olfactory bulbs with rest-of-brain mass vary? Do this by plotting the mass of each structure, as the dependent variable, against rest-of-brain mass, as the independent variable.
2. Next, do you see any differences in scaling between species? Does this affect the slope or intercept? Can you suggest a reason for these patterns?
3. In these scaling analyses, why do we use rest-of-brain rather than total brain size?

**Method for Sectioning and Staining Brains**

1) Turn on the electric heater to melt Gelatine (about 5 mins)

2) Lay the different brain structures (neocortex and cerebellum) with their **medial surfaces down** on your dissection board. Use the diagram below as a guide to **cut it transversely into 2 to 5 mm thick slices** with the blades provided. As you cut the slices lay them out in order in rows on your dissection board with the first section on the left, with the **rostral side facing down**, the **dorsal surface towards the top of the board** and with the **medial surface running vertically.**

3) **Stick the sections down on the black plate for staining**.
- Use the paintbrush to apply a thick layer of melted gelatine, near the top left corner of the plate, **just large enough to be covered by your first section**.
- Blot the underside of the section on a tissue, orient it with **medial surface vertical** **and dorsal surface at the top** then press it down onto the gelatine.
- Repeat with the remaining sections laying them out in neat rows on the plate.
- Allow a few minutes for the gelatine to harden by cooling.

4) **Stain the sections using modified Mulligan's technique** [see Green (1937) *J. Anat.* 67]
a) Place plastic plate with sections into plastic tray arranged with drain hole positioned over the sink.
 - Fill tray with water to rinse sections briefly, then drain by tipping water into sink
 - Cover the sections with 2% tannic acid for 1 minute.
b) Carefully empty the tannic acid solution **into the collection vessel**
 (**DO NOT** empty the tannic acid down the sink).
c) Place the tray back over the sink and wash sections in running tap water for 3 minutes.
d) Cover the sections with 0.4% iron alum until grey matter **turns pale blue-grey**. **It is important not to over stain (ask a demonstrator to watch the process)**
e) As soon as staining is sufficient, pour off iron alum and flood with tap water to stop the staining.
f) Cover sections with 5% bleach for 0.5 to 1 minute until the blue colour becomes grey.
g) Wash in running water for 1 to 2 minutes.

5) Blot the sections and dry the under surface of the plastic plate using tissue**.**

6) Make sure all the sections are labelled with a number starting at 1 and going up to the maximum number of sections for that brain structure. Then use a ruler to record the depth of each section. Record the depth of each numbered section in the online spreadsheet

7) With a ruler in shot, take (or access online) three photographs, one of the stained neo-cortex sections, one of the stained cerebellum sections and one with all the stained sections present.

The ruler will be required to provide scale when we use computer software called Fiji - Image J to calculate the areas and when combined with the depth of the sections the volumes of these sections.

**Analysing and Comparing Brains**

Download the photos you have just taken of your brain sections, a demonstrator will help you do this. Open these one at a time in Fiji – Image J. You can use the transform tool to rotate the image and the zoom and hand tools to move the image around.

Once you have an imported image you can set the scale using the **analyse and set scale features** by drawing a line along the ruler (the longer the better). Now you have the scale set in mm you can use the polygon tool to calculate the area of each total section. You will also need to measure the thickness of each section (to nearest 1mm) to multiply the polygon area by thickness to get a volumetric measurement. Log this in the shared spreadsheet.

You next need to record the separate volumes of grey and white matter for each section. Firstly, record the total area if the section and then the white matter area. Secondly, subtract the white matter area value from the total area value to get the grey matter area. Record these values in the your spreadsheet and the class data spreadsheet. Do this for both hemispheres (and both species) of the neocortex and cerebellum.

Now analyse the data in the spreadsheet to answer the following:

Using the raw volumes:

1. Compare the proportions of the neocortex and cerebellum that are grey matter (for both species separately).

Next, examine the scaling relationships between white and grey matter using log-transformed volumes:

1. Does white matter scale with grey matter with isometric, hypo-allometric or hyper-allometric scaling? Plot separate figures for the sheep and pig brain. Do both species scale in the same way?
2. Based on the scaling relationships identified in (2), how does the proportion of each structure that is white and grey matter vary as brains increase in size?

**Conclusions**

Summarise the key differences between sheep and pig brains and suggest biological reasons for these. Do you find any evidence for adaptive shifts in brain structure between species?

Do your results support predictions about how brain structure volume affects the relative amount of long-range axons in the brain?

Evaluate the reliability and accuracy of both the method and your conclusions.

*Did you find the expected scaling relationship if so, can you explain why we expect to see this pattern?*

*If you did not find the expecting scaling pattern can you suggest reasons for this (consider the experimental method and source of the brain material)?*
