# Supplementary material for: A Novel Practical Session to Teach Concepts of Allometric Scaling of Brain Structures to Undergraduate Students Using Vertebrate Brains
Source: J Undergrad Neurosci Educ. 2025 Dec 31;24(1):38–46. doi: 10.59390/001c.154559 (PMC13127676; doi:10.59390/001c.154559)
Supplement: Appendix 3 [file junejournal_2025_24_1_154559_322905.docx]

**Appendix 3: Technical Preparation Sheet.**

The following is the technical prep sheet used when preparing for this session. Students work in pairs and so quantities must be scaled up depending on the class size.

**Consumables/Chemicals and Equipment**

All students must be provided with suitable personal protective equipment (PPE), googles, gloves and laboratory coat.

| **Animal/plant material:** | **Notes** |
| --- | --- |
| ½ Sheep brains or ½ pig brain per pair. Alternate between pairs so all groups can see different brain types. | Sheep brains need to be ordered from Blades Biological. Pig skulls purchased from abattoir and extracted as per Appendix 4. Sheep brains were purchased from Blades Biological, product code: PZK 195. They are preserved in ***Carolina’s Carosafe*** which is a formaldehyde-free holding solution. It has quite a strong scent and is still hazardous despite having no formalin, so the brains should be washed for 48 hours before use. They have the dura matter still attached, this must be removed in a fume hood before washing. |

| **Equipment and chemicals (per pair)** | **Notes** |
| --- | --- |
| Large white tray |  |
| Forceps x 2, scissors, probes, spatula, scalpel | For dissection |
| Large grey plastic plates for sections (must fit trays) | 27.5cm/20.5cm |
| Plastic chopping board | For sectioning |
| Bottle of 2% tannic acid 800ml | Use dark pre labelled bottles |
| Bottle of iron alum 800ml | Use dark pre labelled bottles |
| Bottle of 5% bleach solution 800ml | Can use Duran bottles or conical |
| Microtome blade | We use Epredia™ MX35 Premier™ Disposable Low-profile Microtome Blades, purchased from Fischer Scientific, product code: [10687875](https://www.fishersci.co.uk/shop/products/mx35-premier-disposable-low-profile-microtome-blades/10687875). These are single use blades and should be collected into a sharps bin at the end. |
| Brain image laminates | These are photographs of different brain structures visible in the different sections moving through the brain in a rostral to caudal progression. Could be replaced my text books or similar. |
| Paint brush | For application of gelatine |
| Stopwatch |  |
| Ruler (needs graduations right to the edge to measure slice depth) |  |

| **Equipment to be shared between 2-4 pairs depending on availability** | **Notes** |
| --- | --- |
| Heat lamp and gelatine dish | Set out between pairs (Usually 2 on a bench of 6 seats), need to be turned on at least 20mins before practical to melt the gelatine |
| Balances and weighing boats | All available, 2dp & 3dp |

| **Equipment available in laboratory** | **Notes** |
| --- | --- |
| Photographic trays with “T” connectors & rubber tubing | At every available sink enable. |
| Funnel | One with every photographic tray |
| Paper towels | Several on tops of benches |
| Gloves | Check racks are topped up |

**Solutions – approx. 800 ml of each per pair:**

- 0.2% iron alum (Ammonium Ferric Sulphate). Dispense into dark glass bottles and wash out to avoid glassware becoming stained. Solution made with Ammonium Iron (III) sulphate dodecahydrate purchased from VWR, product code: 21094.233. Mix on magnetic stirrer, make no more than the day before and dispense into dark glass bottles.
- 2% Tannic Acid. For 10 L , mix on stirrer, carefully add small amounts a bit at a time to avoid clumping. Make fresh or day before. Dispense into dark glass bottles and wash out to avoid glassware becoming stained. Tannic acid purchased as a dry powder from Sigma-Aldrich, product code: 403040. Mix on a magnetic stirrer, carefully add small amounts a bit at a time to avoid clumping. Make fresh or day before. Dispense into dark glass bottles as it will stain normal glassware. Ecotoxin so dispose of it as in waste disposal section below.
- 5% Thick Bleach. Make with any unscented thick toilet bleach, we used the Jangro Professional Thickened Bleach, product code: BC015-5. Try to avoid making excessive amount of bubbles when mixing, dispense into glass bottles or conical flasks. This must be made with thick bleach, not thin. It has been tested with several brands of thin bleach over the years of running this session and it was found that the staining quality is not as good with thin bleach

**Gelatine/Glycerine mix:**

Gelatine/glycerol mix, this is made using powdered Gelatine from VWR, product code: 24350.262 and Glycerol from Fischer Scientific, product code: 10579570

- 107g gelatine
- 107 mls glycerine (glycerol)
- 500 mls d.H_2_O (pre-boiled in kettle for quickness) place in 500ml beaker & add gelatine a bit at a time to avoid clumping.
- When dissolved (might require further heat), add glycerine. Pour in to evaporating dishes and store in fridge. Can make extra into beaker and cut out chunks to top up.

Note: 1L was enough for 3 classes of 20 groups – but only using 12 ish bowls

**Collection of brains:**

The sheep brains are ordered in advance from Carolina Biological Supply, through Blades Biological. The pig brains are collected by the tech team and extracted (see Appendix 4) over the summer and preserved in formalin ready for the Spring practical. To collect brains as per the instructions in appendix 4 the technician must have suitable PPE and equipment to include scrubs, wellies, large apron, anti-cut gloves, nitrile gloves, googles, a work surface with a drain, knives, large spiked vice, large hack saw, wide head chisel, hammers, scalpels, fine and blunt forceps, razor blades, bone snips and suitable cleaning supplies and wsste disposal stream for left over tissues such as Virkon. Must have suitable waste collection for animal tissue.

**Technical preparation of the extracted brain material**

All brains need to have meninges removed and be washed for at least 48 hours before the practical to remove most of the formalin. Longer or multi-session laboratory sessions could let student remove the meninges but this is time consuming.

**Labelling:**

The students need the brains numbered so they know what they have when inputting their data. Each grey board has a small piece of tape labelled in the following way: S1R, S1L, P1R, P1L, where by S or P represents “Sheep” or “Pig”, the number denotes which brain it came from and R and L represent “left” or “right”. This enable student to pair the data from their brain hemisphere with the other hemisphere analysed with another group. This data can all be pooled in the final class datasheet.

An example of how the equipment looks for each student pair at the start of the laboratory can be seen in figure 1.


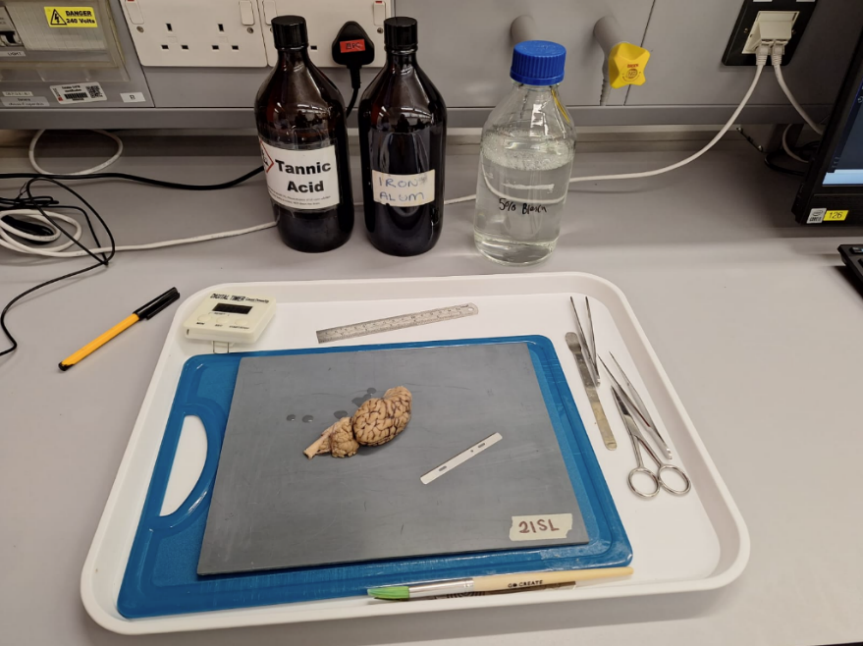


Figure 1: An image of the technical set up for each pair when students arrive. The other equipment required for staining in the sinks is not shown.

**Waste disposal:**

Due to the hazardous nature of chemicals used in this session, waste disposal must be completed in accordance with local guidelines, the waste disposal guidelines are as follows:

| **Waste** | **Disposal Method:** |
| --- | --- |
| Animal tissue, bone and brain matter | Hazardous waste, must be sent for incineration via licensed local waste stream. Can be frozen in interim to prevent decaying matter being left out |
| Sharps: microtome blade and scalpel blades | Collected into a sharps container and sent for incineration |
| Gelatine/glyverol mix | Once set the solid jelly can be removed from the dish and placed into a hazardous waste bin for incineration. |
| Bleach solution | Can be washed down the drain with extra water |
| Iron alum solution | Can be washed down the drain with extra water |
| Tannic acid solution | Must NOT enter the drains, serious ecotoxin. Waste must be collected into appropriate containers and disposed of via a licensed waste disposal company |
