# Supplementary material for: A Novel Practical Session to Teach Concepts of Allometric Scaling of Brain Structures to Undergraduate Students Using Vertebrate Brains
Source: J Undergrad Neurosci Educ. 2025 Dec 31;24(1):38–46. doi: 10.59390/001c.154559 (PMC13127676; doi:10.59390/001c.154559)
Supplement: Appendix 4 [file junejournal_2025_24_1_154559_322904.docx]

**Appendix 4: Sourcing and extraction of pig brain material**

Whilst vertebrate pig brain material can often be sourced pre-isolated, cleaned and stored in formalin, one does pay a financial premium for this service, the brains can be in varying levels of preservation (e.g. some have been damaged in preparation) and supply can be limited (particularly for pig brains) we extracted and prepare pig brains in house.

**Initial sourcing of animal material:**

The Bristol Veterinary School has an Abattoir (Langford) and facilitated the purchase of pig heads (£15 per head) from a local farmer sending his animals for slaughter.

**Materials:**

- Personal protective equipment – scrubs, wellies, large apron, anti cut gloves, nitrile gloves, goggles
- A work surface with a drain/way to collect liquids
- Sharp knives
- A large spiked vice – it must be large enough to hold a pig head and spiked to prevent the head moving when being cut
- A large hack saw with a blade capable of cutting bone
- Wide head chisel
- Hammer
- Scalpel
- Fine and blunt forceps
- Razor blade
- Bone snips
- Bags/containers to take the brains away in
- Suitable cleaning supplies and waste disposal stream for left over tissue – we used Virkon and the waste collection was facilitated by a licensed waste company.
- 5% formalin
- Hand Held Oscillating Saw
- Strong pliers
- Chopping board
- Fume hood

**Method:**

1. Place a head on the work surface and using a knife, remove as much excess tissue, such as the ears and skin, from around the top of the skull as possible. The winged top of the occipital bone must be exposed.
2. Place the hack saw on the outside of the winged top, but close to the base. Saw down at a slight inward angle, stopping just before hitting the brain cavity. This is usually only about 1.5-2cm deep. Repeat on the other side of the winged occipital bone. See Figure 1A, labels 1 & 2.
3. At the back of the head make two more cuts, joining the first two. They should be angled downwards towards the hole at the base of the skull where the brain stem should be visible. Figure 1B, labels 3 & 4.
4. Finally make a cut straight across the front of the skull, joining on each side with the first two cuts. Figure 1A, label 5.


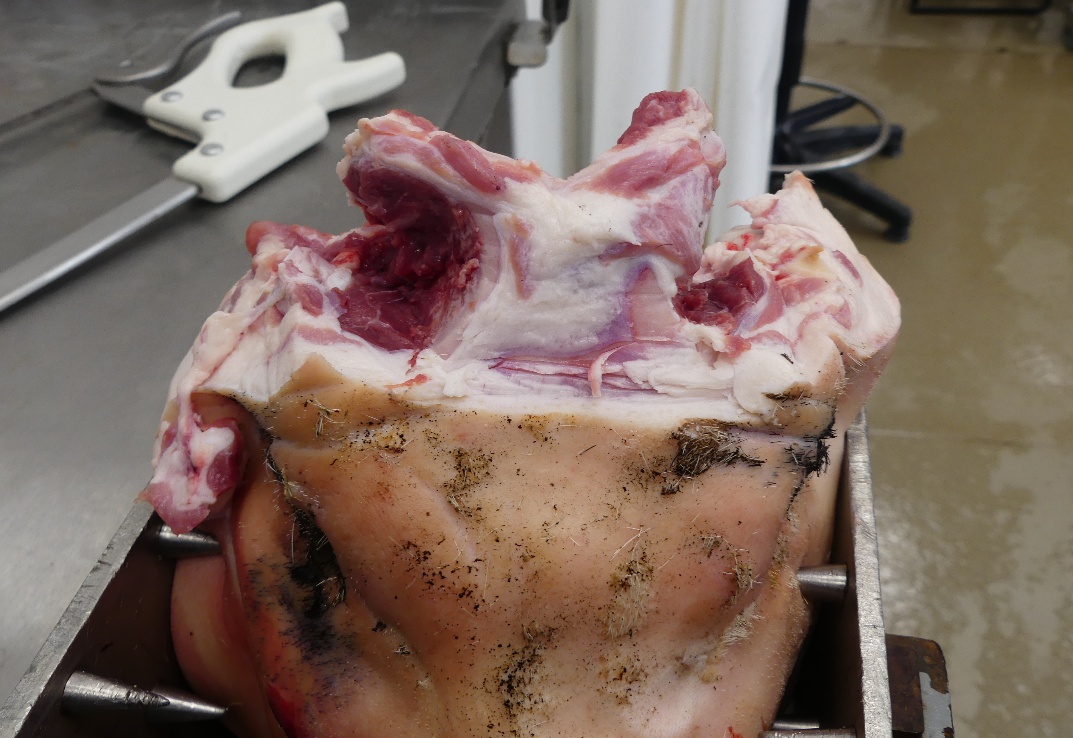

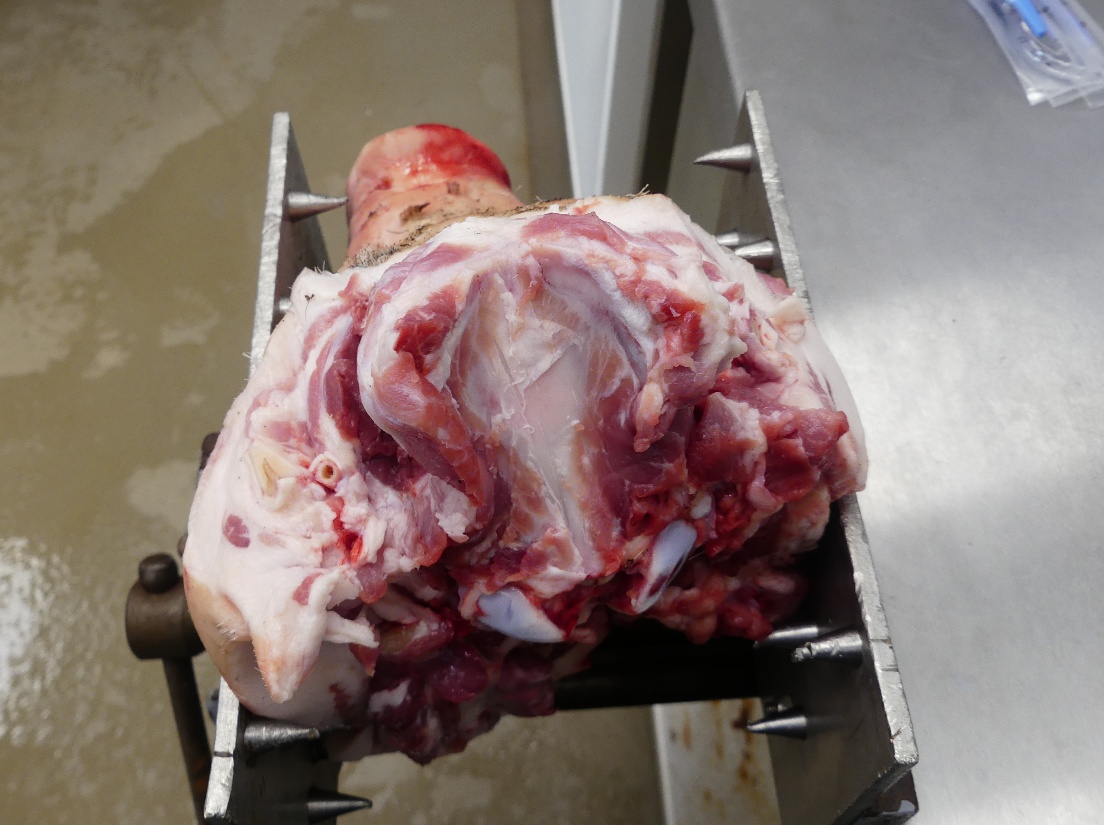


**A**

**B**

**3 & 4**

**5**

**1 & 2**

Figure 1. A) Rostral view of the pig head. Winged top of occipital bone visible at the top (center of the image). More in depth description of what we are looking at and the cuts taking place. B) Caudal view of pig head. Green line indicates the angled cut lines for the hack saw. These cuts should join the cuts made in 1A. All cuts should be no deeper than 1.5-2cm into the bone. This should be deep enough to access the brain without cutting into it.

1. Next take the chisel and hammer, insert the chisel into the cuts and use the hammer to break the final connecting pieces of bone. Leveraging the chisel outwards from the head can help to break connecting pieces (see Figure 2).
2. Insert the chisel into the cut 5, seen in Figure 1A, as deeply as it can go without being pushed into the brain, and push down. This should begin to leverage the top part of the skull up and off. You may discover places where you now need to break pieces of the skull bone with the chisel or cut connective tissues with the scalpel.
3. An entire piece of the skull should lift up and backwards. It will most likely still be connected near the brain stem by various connective tissues, carefully cut these with a scalpel and entirely remove the top piece of skull.


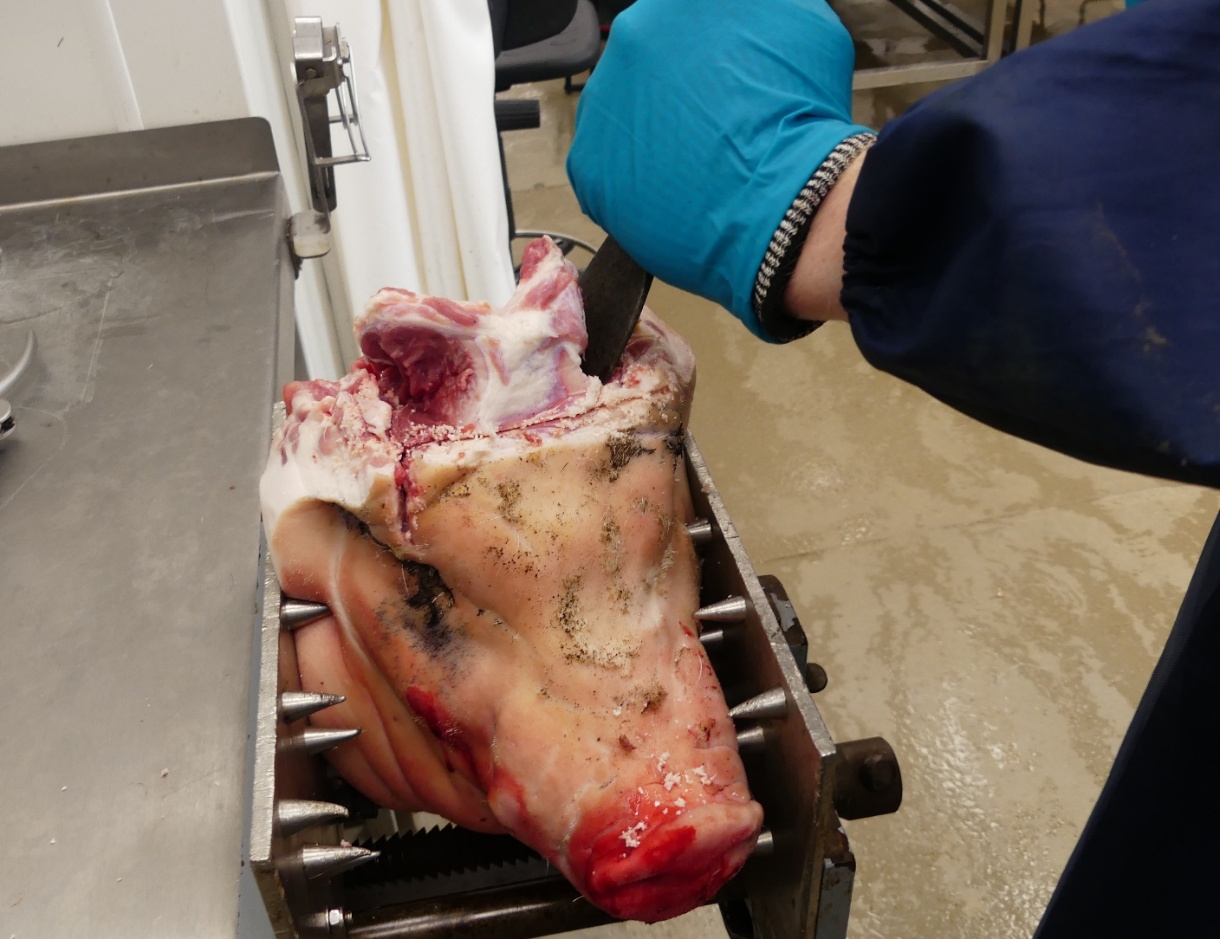


Figure 2: Use of chisel and hammer into the original hacksaw cuts to break the bone.

1. You should now have the top of the brain exposed, this can now act as a guide regarding the position and depth of cuts which will help remove the brain without damage.
2. Take the hack saw and saw straight down either side of the brain, as close as you can without cutting into the brain. You must go deeper than the brain and the olfactory lobes which will sit slightly below the rest of the brain, by approximately 1.5-2cm. See figure 3 for details.
3. Cut directly under the brain from the front of the head, usually below the eyes will ensure the olfactory lobes will not be hit. Figure 3 for approximate position.


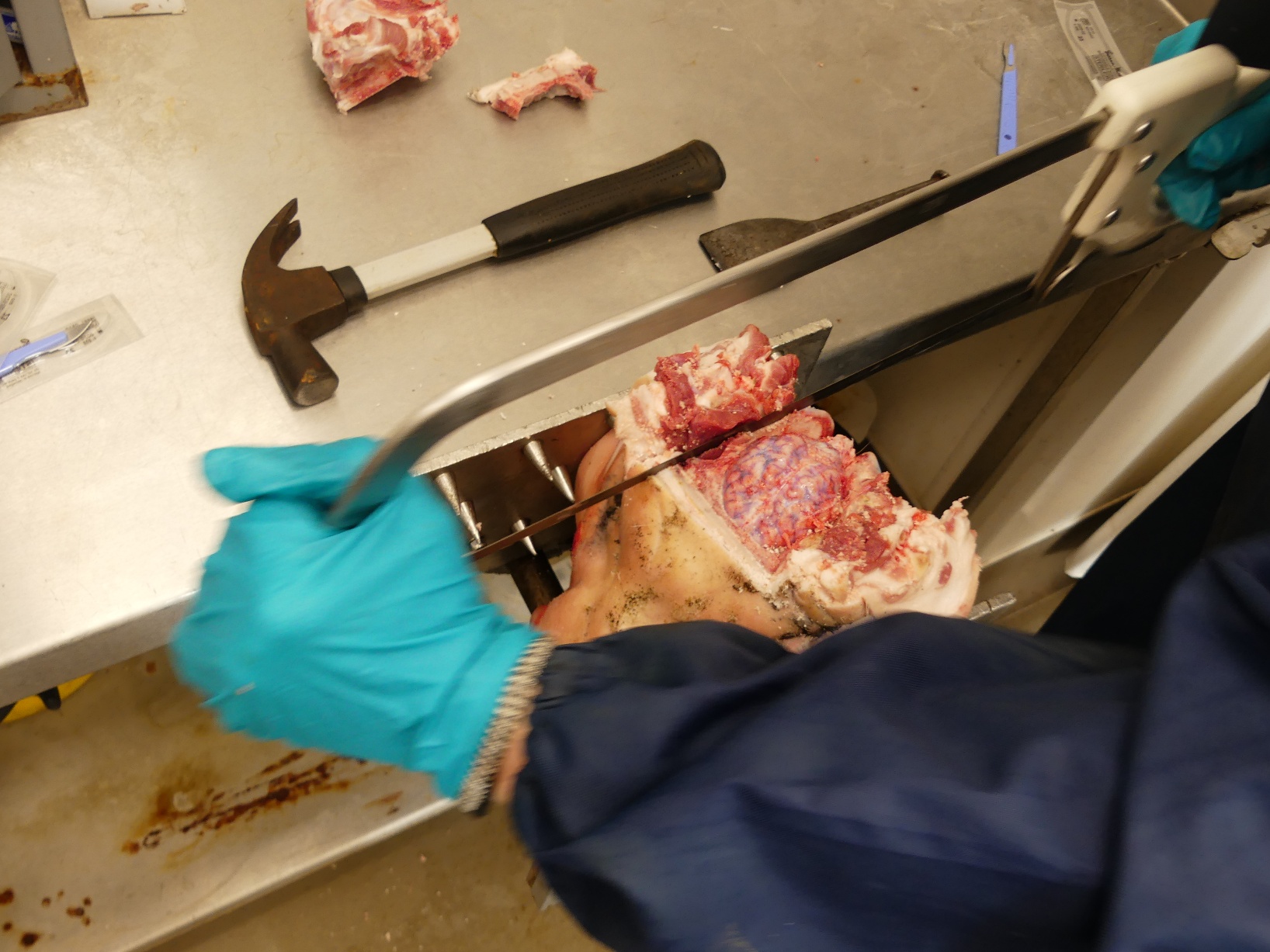


Figure 3. Almost Parallel yellow lines indicate hack saw cut lines running very close to the brain (indicated by the black arrow). Single yellow line indicates approximate location for a cut into the rostral face of the pig head.

1. Once you have sawed most of the way through the head, remove the saw and insert the chisel. Push down to begin lifting the section of head containing the brain up and away from the rest of the head. You will need to cut various connecting tissues with a scalpel as you go. See Figure 4 for guidance.
2. Once all the connective tissues are cut the section should just lift out. You should now have the brain, with the top exposed and the sides, front, back and underside still encased in a small layer of bone and tissue. See Figure 4 for guidance


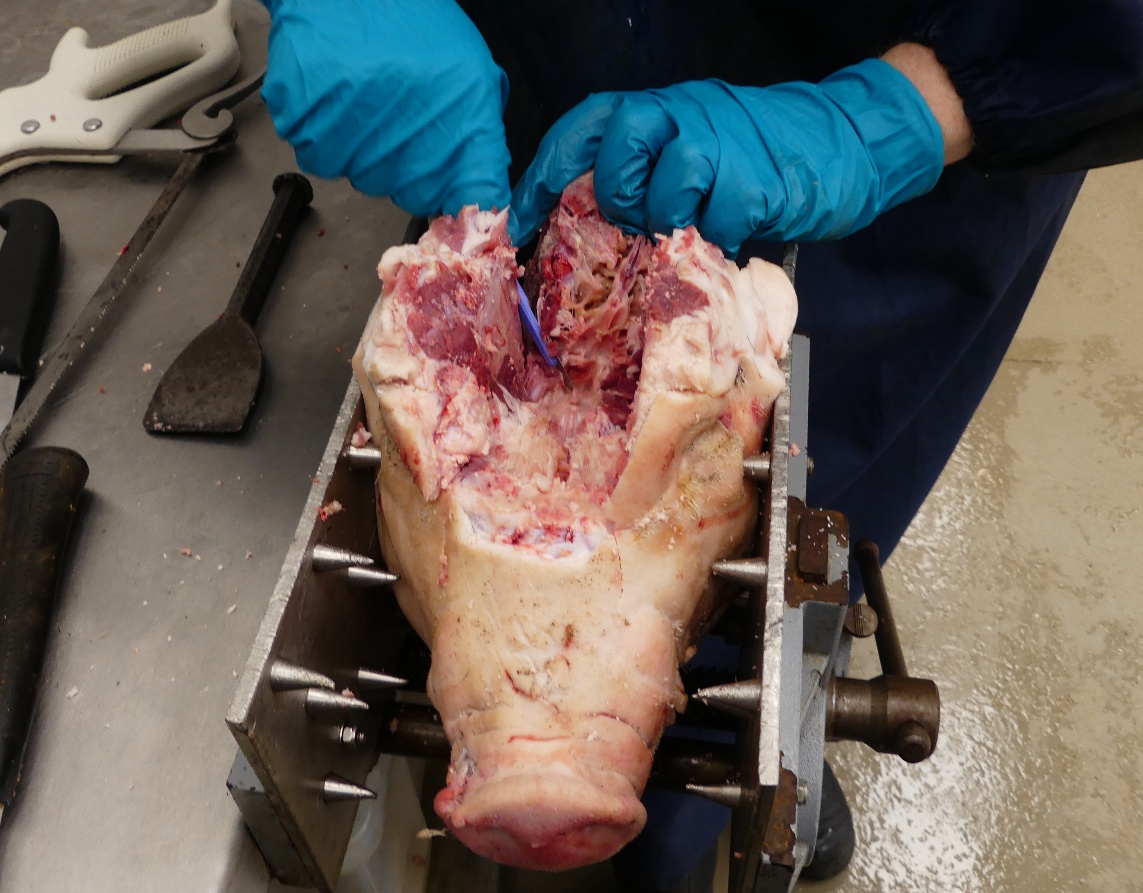

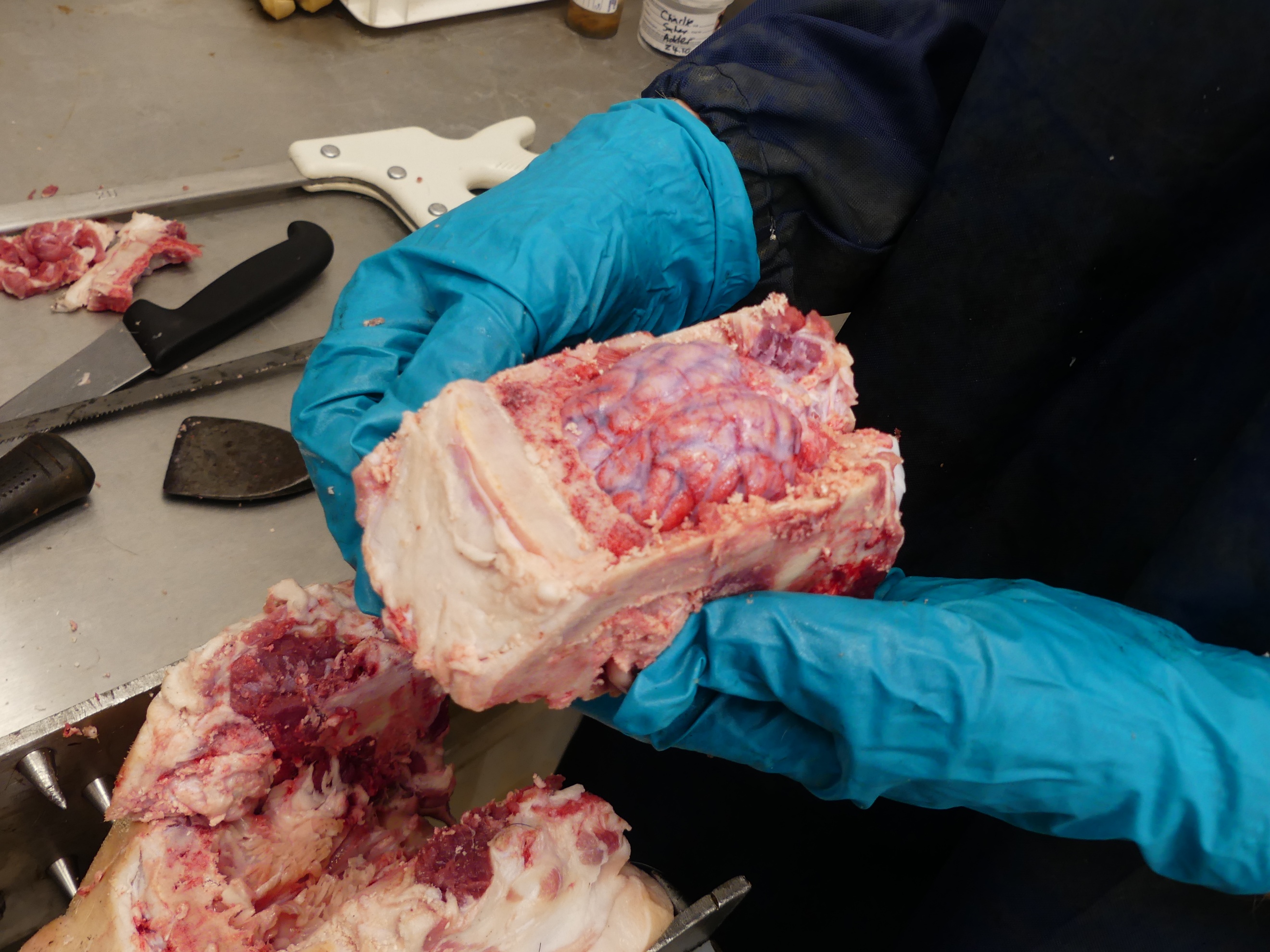

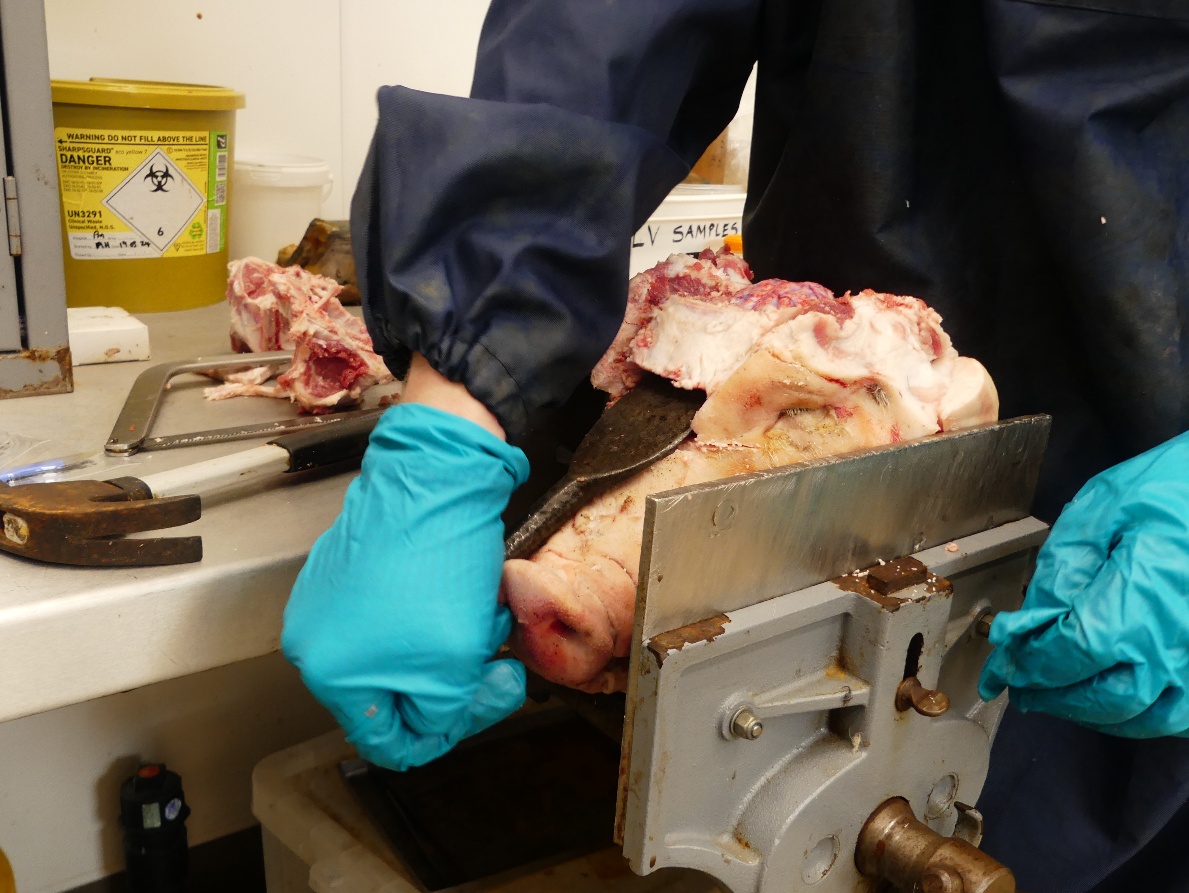

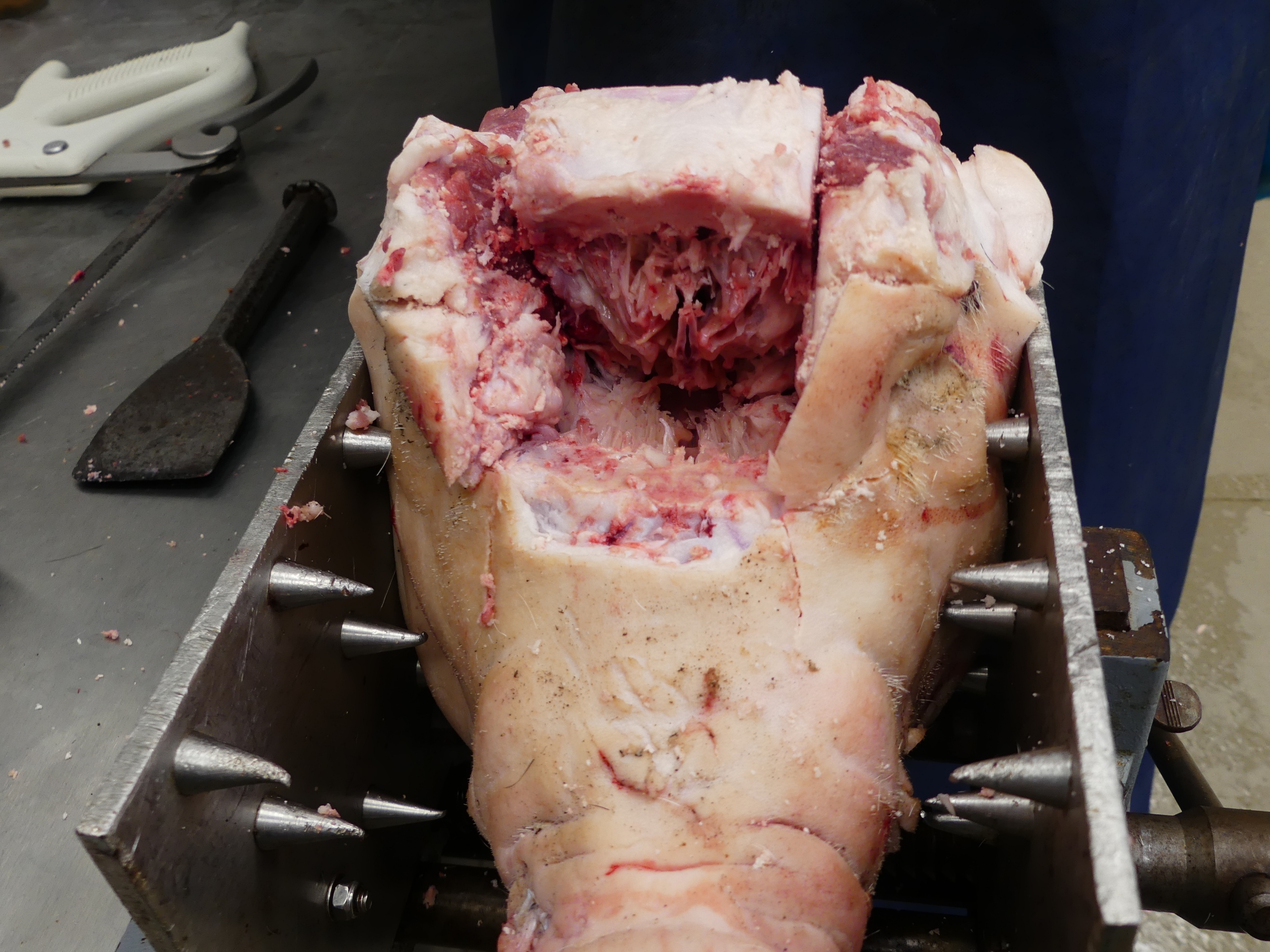


**A**

**B**

**C**

**D**

Figure 4. A) Use of chisel into the rostral cut of the pig head. Downwards pressure applied to lift up pig head material. Remove connective tissues with a scalpel as necessary. B) Rostral view of pig head after removal of living material in step 11 (figure 4A). C) using a scalpel to remove connective and other tissues to isolate the section containing the brain. D) Brain, meninges and some other tissues removed as a single block.

1. Place this into your appropriate container to take it away with you, and repeat the previous steps if you have more than one head.
2. Next, take your prepared brain surrounded by tissue and bone. Using fine forceps, carefully lift the dura mater away from the surface of the brain. Take great care, the brain is very soft and can be easily stabbed at this point with the forceps. Using a scalpel or razor blade carefully make at least 3 cuts, at least 1 cm long into the dura mater without cutting the brain itself. This will allow the formalin to permeate the brain.
3. Once done, submerge the whole piece in 5% formalin and leave it covered for at least two weeks to fix the tissue. This can be left for longer. We have stored for 5 months with no problems before moving to the next stage of brain extraction.
4. Remove the brain chunk from the formalin two days before starting. Place it in a continually running bucket of water for at least six hours. Then continue to soak it, changing the water every few hours until ready to use. This removes most of the formalin, making it safer to now work with.
5. *Sawing through the bone can cause a large amount of fine bone dust to be created, therefore it is essential that the following method is completed in a fume hood to avoid inhalation of the bone dust.*
6. Take the washed brain chunk out of the water bucket and place it in the fume hood, on the chopping board.
7. Using the oscillating saw, carefully cut small chunks of bone away down one half of the brain, to expose one side of the brain. The pliers can be used to snap off cut pieces (see figure 5). The brain will be much firmer and easier to handle now, however still take care to not go all the way through into the brain.
8. Slowly remove small pieces of skull, larger pieces are more difficult to snap off without damaging the brain.

**B**

**A**


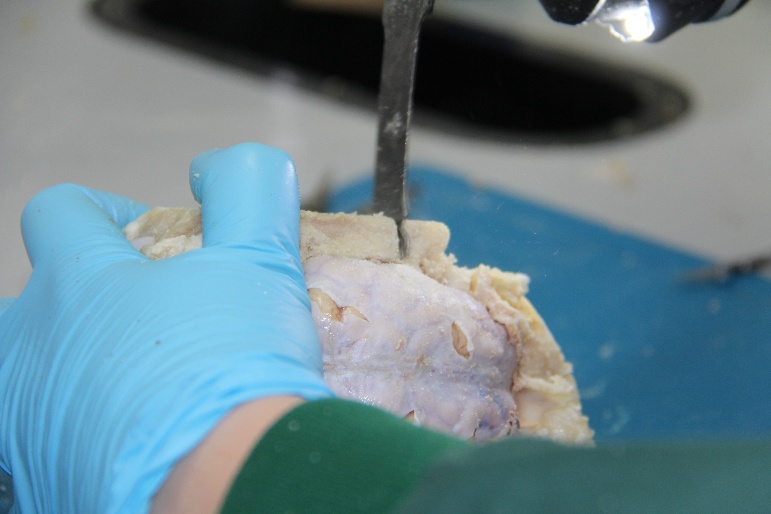

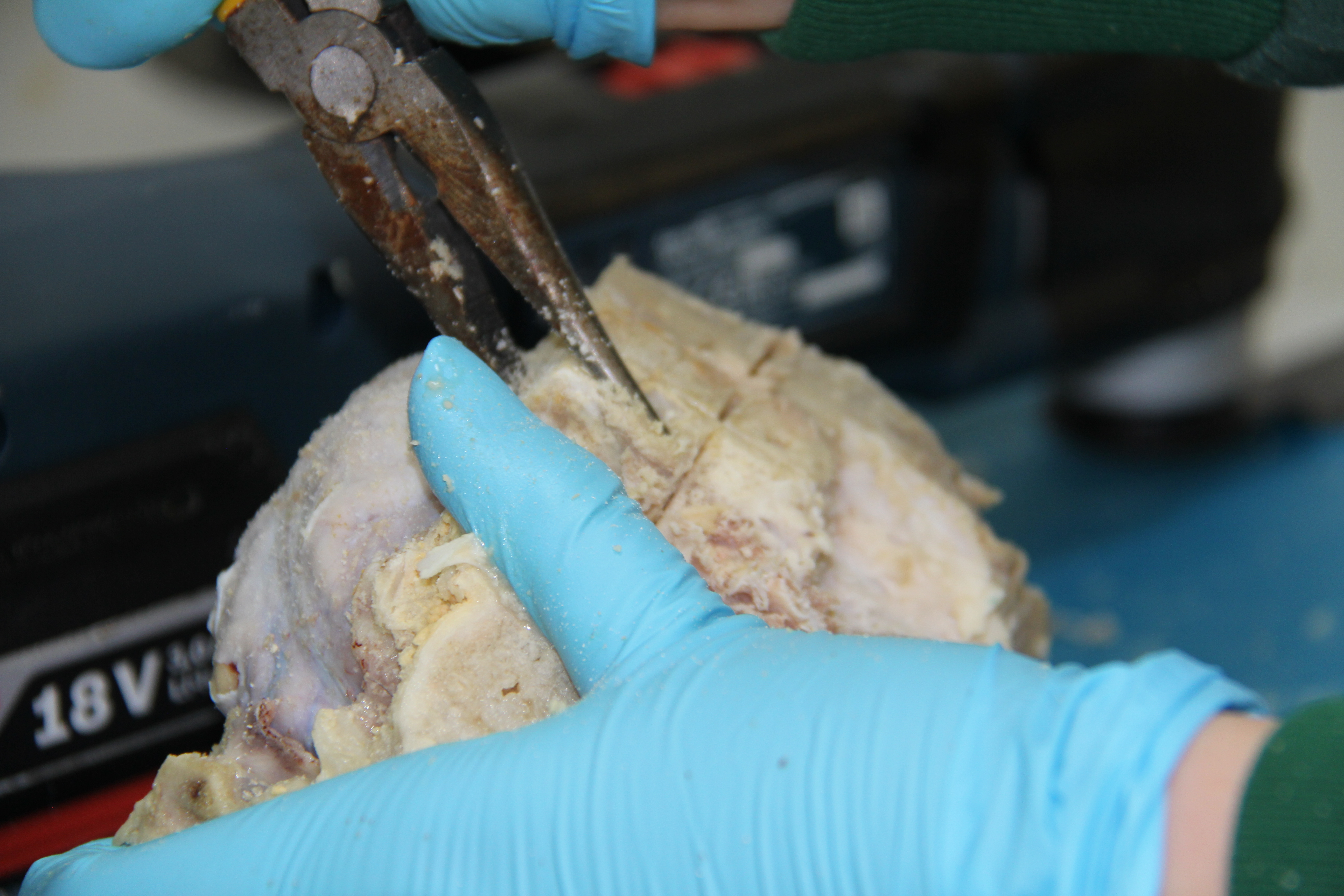


Figure 5. A) Use of oscillating saw to careful remove small pieces of tissue. B) Removal of tissue using pliers.

1. When the side of the brain is exposed the dura mater can be carefully cut with a combination of razor blades and scalpels, then removed using forceps to carefully lift it off the surface of the brain
2. Carefully use your finger to lift the back of the brain under the brain stem. There are various connecting tissues that must be cut, including the optic nerves which can be seen as two thicker cream bands that cross underneath the brain, between the brain and the inside of the dura mater. Figure 6 for detail.
3. Carefully cut all these connecting tissues without lifting the brain too high. If the brain is lifted too high, it can cause the olfactory bulbs to be ripped off as they are connected to the rest of the brain by a small amount of easily torn tissue.
4. The olfactory bulbs are found at the rostral end of the brain and will be attached to the nasal cartilage. Do not pull on them or the brain, they are very delicate.
5. Using the bone snips, scalpel and saw if necessary, dissect out the cartilage around the lobes to expose them. Using the scalpel handle to carefully ease in underneath the lobes, they be carefully lifted out.
6. The brain with the attached lobes should now be free of the chunk of bone and tissue.
7. This can now be placed back into formalin to remain preserved until ready for use


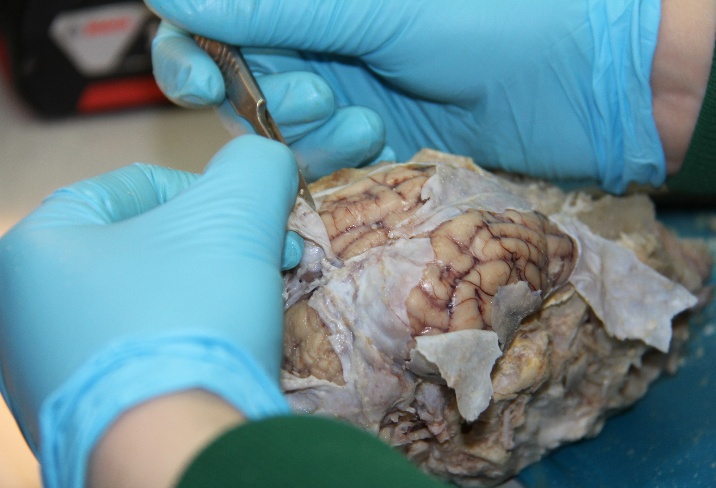

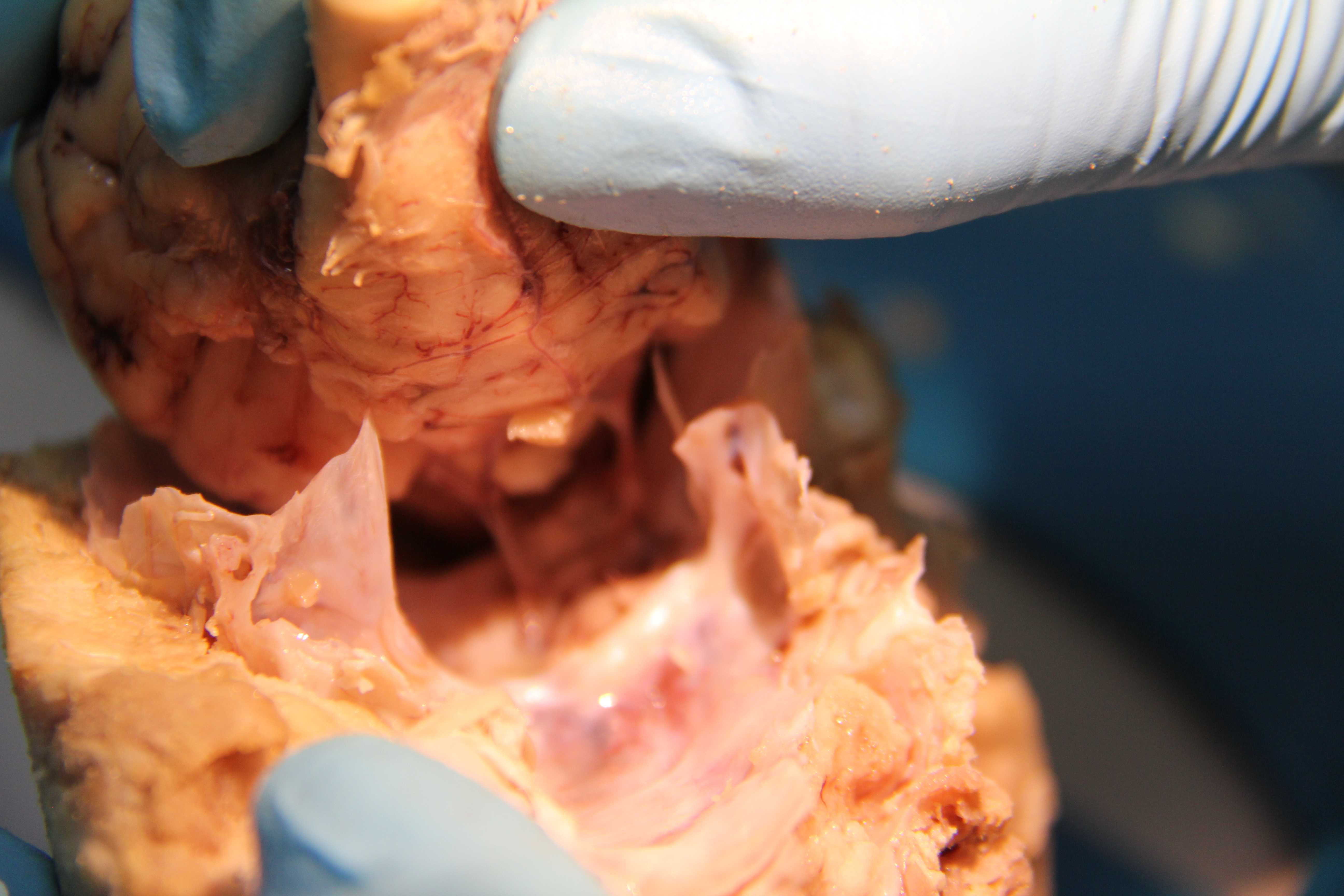

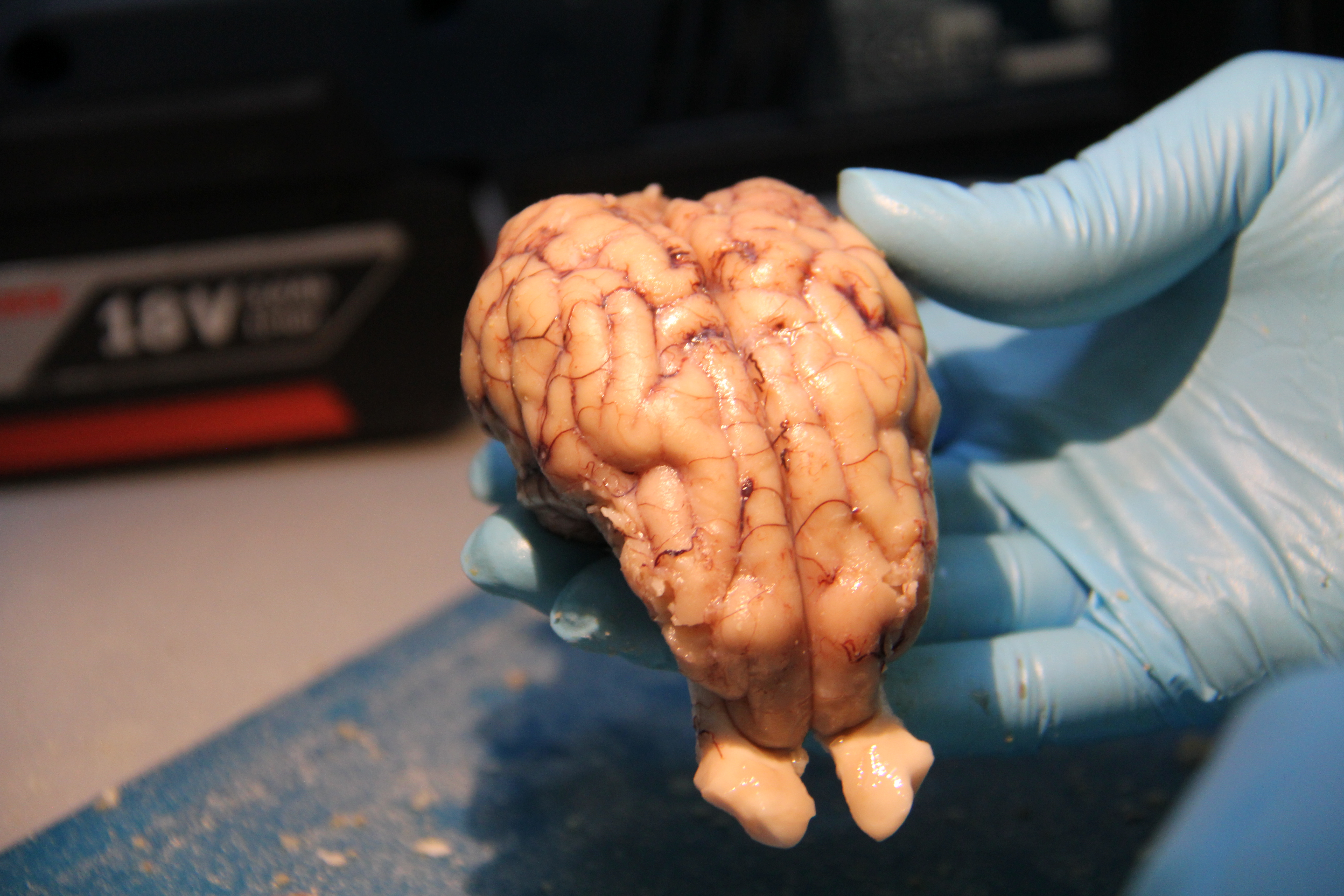


**A**

**B**

**C**

Figure 6: A) Use of scalpel and forceps to carefully remove the dura mater and meninges (step 21). B) Lifting of the brain in preparation to cut the optic nerves and connective tissue (steps 22 and 23). C) Final removed brains with perfectly intact olfactory bulbs visible in the lower part of the image.

*Technical guide produced by Kelly Cavaciuti, Teaching Technician at the School of Biological Sciences, University of Bristol.*
