# Supplementary material for: A Novel Practical Session to Teach Concepts of Allometric Scaling of Brain Structures to Undergraduate Students Using Vertebrate Brains
Source: J Undergrad Neurosci Educ. 2025 Dec 31;24(1):38–46. doi: 10.59390/001c.154559 (PMC13127676; doi:10.59390/001c.154559)
Supplement: Student Survey (Part I) [file junejournal_2025_24_1_154559_322907.pdf]

Evaluating Novel Methods For Teaching Students About Scaling Relationships in Brains PART 1

We (Stephen Montgomery and Christopher Cammies) are conducting a study that evaluates the effectiveness of the "vertebrate brains" practical for teaching students about scaling relationships in brains.

The study has 2 surveys. The first of which, you are accessing now, is designed to test your baseline understanding of 10 neurobiology concepts. You can demonstrate this by trying to answer correctly (to the best of your ability) the 10 enclosed neurobiology questions.

The second survey will take place after the completion of the "vertebrate brains" practical session and will re-test your understanding of 10 neurobiology concepts and ask a further 10 questions examining your subjective experience of the practical session as a learning experience.

On completion of the second survey you will get the chance to be entered into a random prize draw with 4 opportunities to win a £50 Love2Shop (or equivalent value) voucher.

By completing this survey (clicking 'Submit'), you are giving your consent and confirming that you agree with the following statements:

- I agree to take part in the survey, having read and understood the information given in the Participant Information sheet.

- I understand and acknowledge that this project is designed to promote scientific and pedagogic knowledge and that the University of Bristol will use the data I provide for no purpose other than research.

- Your participation is entirely voluntary, and all information gathered will be completely anonymous. To this end, the research team will maintain the confidentiality of the data you provide by not divulging identifiable information on individuals. Your anonymity is assured in conducting this research and in the research outputs I publish.

- I understand that any information provided will be securely held, processed, and may be utilised in future analysis. I understand that these data will be used only for the purpose(s) set out in the information sheet, and my consent is conditional upon the University complying with its duties and obligations under the Data Protection Act / General Data Protection Regulation (GDPR).

- I understand that after the study period, the data will be made "open data". I understand that this means the anonymised data will be publicly available and may be used for purposes not related to this study; however, it will not be possible to identify me from these data.

If you would like any further information or have concerns about this research project, feel free to contact Christopher Cammies (cc15486@bristol.ac.uk). Should you wish to make a complaint regarding this work and its delivery, please contact the University of Bristol Research Governance Team (research-ethics@bristol.ac.uk).

Thank you for taking part in this research.

Testing of Neurobiology Concepts

1. In order for us to anonymously pair your pre-session and post session understanding of these neurobiology concepts we will need a unique identifier to track your scores. Please create a unique identifier with at least 4 regular character, 2 regular numbers and 2 unique symbol characters (e.g. * or $).

   YOU NEED TO RECORD THIS CODE SOMEWHERE SO YOU CAN USE IT AGAIN FOR THE SECOND SURVEY

   Write out your unique identifier code here:

2. White matter contains: *

- ◯ A higher proportion of long distance, myelinated axons than grey matter
- ◯ A similar proportion of long distance, myelinated axons than grey matter
- ◯ A lower proportion of long distance, myelinated axons than grey matter

3. Electrical communication has benefits over chemical communication because it is: *

- ◯ Energetically cheaper
- ◯ Faster
- ◯ Requires less cellular "kit"

4.
A 'grade-shift' in scaling occurs when: *

- ◯ The slope of the scaling relationship differs between groups
- ◯ The intercept of the scaling relationship differs between groups
- ◯ The groups share the same scaling relationship

5. You can speed up electrical signalling by: *

- ◯ Increasing the diameter of a neuron
- ◯ Elongating axon length
- ◯ Removing myelin

6.
If the natural log of a particular component scales with a slope of 1 against the natural log of brain size which type of scaling is taking place *

- ◯ Hypo-allometric
- ◯ Isometric
- ◯ Hyper-allometric

7. The main, continuous energetic cost of a neuron is related to: *

- ◯ Firing an action potential
- ◯ Maintaining resting membrane potential
- ◯ Maintaining the cell membrane

8. The cocktail party problem referes to the challenge of *

- ◯ Finding food in a complex environment
- ◯ Perceiving sounds in noisy social settings
- ◯ Keeping track of the social interactions in your group

9. When the slope of a scaling relationship (beta) is above one that is *

- ◯ Isometry
- ◯ Positive allometry
- ◯ Negative allometry

10. Electric fish have one particularly enlarged brain region, which one: *

- ◯ Olfactory bulbs
- ◯ Cerebella
- ◯ hippocampus

11. Across mammalian species, what type of scaling relationship would you expect to see between the proportion of white to grey matter and brain size (note: you may not have seen this trend in the practical). *

- ◯ Hyper-allometry e.g., the volume of white matter increases more than the volume of grey matter as brain volume increases
- ◯ Isometry e.g., the volume of white matter and grey matter increase proportionally with total brain volume
- ◯ Hypo-allometry e.g., the volume of white matter increases less than the volume of grey matter increases as brain volume increases

---

This content is neither created nor endorsed by Microsoft. The data you submit will be sent to the form owner.

Microsoft Forms
