# Supplementary material for: A Novel Practical Session to Teach Concepts of Allometric Scaling of Brain Structures to Undergraduate Students Using Vertebrate Brains
Source: J Undergrad Neurosci Educ. 2025 Dec 31;24(1):38–46. doi: 10.59390/001c.154559 (PMC13127676; doi:10.59390/001c.154559)
Supplement: Student Survey (Part II) [file junejournal_2025_24_1_154559_322909.pdf]

Evaluating Novel Methods For Teaching Students About Scaling Relationships in Brains PART 2

We (Stephen Montgomery and Christopher Cammies) are conducting a study that evaluates the effectiveness of the "vertebrate brains" practical for teaching students about scaling relationships in brains.

The study requires students who have attended the practical session to try to answer correctly (to the best of their ability) 10 neurobiology questions and a further 10 qualitative questions asking about their subjective experience of the practical session on their learning.

This study can be completed as an independent standalone survey (even if you didn't complete part 1) but it also follows on from a first survey that students could have completed prior to the laboratory session. If you completed the first survey the responses to your neurobiology questions will be compared with you previous responses to measure improvements in performance for any of the neurobiology concepts covered.

Completion of this survey will give the participant the opportunity to be entered into a random prize draw with 4 opportunities to win a £50 Love2Shop (or equivalent value) voucher.

By completing this survey (clicking 'Submit'), you are giving your consent and confirming that you agree with the following statements:

- I agree to take part in the survey, having read and understood the information given in the Participant Information sheet.
- I understand and acknowledge that this project is designed to promote scientific and pedagogic knowledge and that the University of Bristol will use the data I provide for no purpose other than research.
- Your participation is entirely voluntary, and all information gathered will be completely anonymous. To this end, the research team will maintain the confidentiality of the data you provide by not divulging identifiable information on individuals. Your anonymity is assured in conducting this research and in the research outputs I publish.
- I understand that any information provided will be securely held, processed, and may be utilised in future analysis. I understand that these data will be used only for the purpose(s) set out in the information sheet, and my consent is conditional upon the University complying with its duties and obligations under the Data Protection Act / General Data Protection Regulation (GDPR).
- I understand that after the study period, the data will be made "open data". I understand that this means the anonymised data will be publicly available and may be used for purposes not related to this study; however, it will not be possible to identify me from these data.

If you would like any further information or have concerns about this research project, feel free to contact Christopher Cammies (cc15486@bristol.ac.uk). Should you wish to make a complaint regarding this work and its delivery, please contact the University of Bristol Research Governance Team (research-ethics@bristol.ac.uk).

Thank you for taking part in this research.

Testing of Neurobiology Concepts

1. Did you attend the practical session?

○ Yes

○ No

2. If you completed the pre-laboratory session quiz then please share your unique identifier code here:

3. White matter contains: *

- A higher proportion of long distance, myelinated axons than grey matter
- A similar proportion of long distance, myelinated axons than grey matter
- A lower proportion of long distance, myelinated axons than grey matter

4. Electrical communication has benefits over chemical communication because it is: *

- Energetically cheaper
- Faster
- Requires less cellular "kit"

5.
A 'grade-shift' in scaling occurs when: *

- The slope of the scaling relationship differs between groups
- The intercept of the scaling relationship differs between groups
- The groups share the same scaling relationship

6. You can speed up electrical signalling by: *

- Increasing the diameter of a neuron
- Elongating axon length
- Removing myelin

7.
If the natural log of a particular component scales with a slope of 1 against the natural log of brain size which type of scaling is taking place *

- Hypo-allometric
- Isometric
- Hyper-allometric

8. The main, continuous energetic cost of a neuron is related to: *

- Firing an action potential
- Maintaining resting membrane potential
- Maintaining the cell membrane

9. The cocktail party problem referes to the challenge of *

- ◯ Finding food in a complex environment
- ◯ Perceiving sounds in noisy social settings
- ◯ Keeping track of the social interactions in your group

10. When the slope of a scaling relationship (beta) is above one that is *

- ◯ Isometry
- ◯ Positive allometry
- ◯ Negative allometry

11. Electric fish have one particularly enlarged brain region, which one: *

- ◯ Olfactory bulbs
- ◯ Cerebella
- ◯ hippocampus

12. Across mammalian species, what type of scaling relationship would you expect to see between the proportion of white to grey matter and brain size (note: you may not have seen this trend in the practical). *

- ◯ Hyper-allometry e.g., the volume of white matter increases more than the volume of grey matter as brain volume increases
- ◯ Isometry e.g., the volume of white matter and grey matter increase proportionally with total brain volume
- ◯ Hypo-allometry e.g., the volume of white matter increases less than the volume of grey matter increases as brain volume increases

13. Answer the following questions below regarding your experience of the practical sessions. *

	Strongly Disagree	Disagree	Neither agree nor diagree	Agree	Strongly Agree
Collecting and analysing data on vertebrate brains during the practical has helped me to critique and evaluate the findings of other researchers investigating scaling relationships in brains (for example identifying limitations in their methodology) better than if I had only learned about this in lectures?	○	○	○	○	○
I would have been able to critique and evaluate the underlying weaknesses in published studies exploring scaling relationships in brains just as well if I had only learned about this in lectures.	○	○	○	○	○
Collecting and analysing data on vertebrate brains during the practical has helped me to understand how different scaling relationships (for brain structures versus total brain size) between species might indicate adaptive shifts in brain structure better than if I had only learned about this in lectures?	○	○	○	○	○
I would have understood how different scaling relationships (for brain structures versus total brain size) between species might indicate adaptive shifts in brain structure just as well if I had only learned about it in lectures.	○	○	○	○	○

	Strongly Disagree	Disagree	Neither agree nor diagree	Agree	Strongly Agree
Investigating scaling relationships as part of an actual experiment in vertebrate brains has increased my interest in studying neuroscience?	○	○	○	○	○
The lecturer(s) should use this practical for future students?	○	○	○	○	○

Subjective Experiences - Part 2

14. How would you rate this practical as a learning experience? *Where 10 is an excellent learning experience and 1 is a terrible learning experience.* *

1	2	3	4	5	6	7	8	9	10

15. Please justify your rating for the learning experience above. *

16. How could the practical session be improved? *

17. Any other thoughts or comments? *

This content is neither created nor endorsed by Microsoft. The data you submit will be sent to the form owner.

Microsoft Forms
